# Assessing COVID-19 Vaccine Uptake and Effectiveness Through the North West London Vaccination Program: Retrospective Cohort Study

**DOI:** 10.2196/30010

**Published:** 2021-09-17

**Authors:** Ben Glampson, James Brittain, Amit Kaura, Abdulrahim Mulla, Luca Mercuri, Stephen J Brett, Paul Aylin, Tessa Sandall, Ian Goodman, Julian Redhead, Kavitha Saravanakumar, Erik K Mayer

**Affiliations:** 1 National Institute for Health Research Imperial Biomedical Research Centre Imperial College Healthcare National Health Service Trust London United Kingdom; 2 Department of Surgery and Cancer Imperial College London London United Kingdom; 3 School of Public Health Imperial College London London United Kingdom; 4 North West London Collaboration of Clinical Commissioning Groups London United Kingdom; 5 National Institute for Health Research Imperial Patient Safety Translational Research Centre Imperial College Healthcare National Health Service Trust London United Kingdom

**Keywords:** health informatics, real-word evidence, COVID-19, medical informatics, vaccine, vaccination

## Abstract

**Background:**

On March 11, 2020, the World Health Organization declared SARS-CoV-2, causing COVID-19, as a pandemic. The UK mass vaccination program commenced on December 8, 2020, vaccinating groups of the population deemed to be most vulnerable to severe COVID-19 infection.

**Objective:**

This study aims to assess the early vaccine administration coverage and outcome data across an integrated care system in North West London, leveraging a unique population-level care data set. Vaccine effectiveness of a single dose of the Oxford/AstraZeneca and Pfizer/BioNTech vaccines were compared.

**Methods:**

A retrospective cohort study identified 2,183,939 individuals eligible for COVID-19 vaccination between December 8, 2020, and February 24, 2021, within a primary, secondary, and community care integrated care data set. These data were used to assess vaccination hesitancy across ethnicity, gender, and socioeconomic deprivation measures (Pearson product-moment correlations); investigate COVID-19 transmission related to vaccination hubs; and assess the early effectiveness of COVID-19 vaccination (after a single dose) using time-to-event analyses with multivariable Cox regression analysis to investigate if vaccination independently predicted positive SARS-CoV-2 in those vaccinated compared to those unvaccinated.

**Results:**

In this study, 5.88% (24,332/413,919) of individuals declined and did not receive a vaccination. Black or Black British individuals had the highest rate of declining a vaccine at 16.14% (4337/26,870). There was a strong negative association between socioeconomic deprivation and rate of declining vaccination (*r*=–0.94; *P*=.002) with 13.5% (1980/14,571) of individuals declining vaccination in the most deprived areas compared to 0.98% (869/9609) in the least. In the first 6 days after vaccination, 344 of 389,587 (0.09%) individuals tested positive for SARS-CoV-2. The rate increased to 0.13% (525/389,243) between days 7 and 13, before then gradually falling week on week. At 28 days post vaccination, there was a 74% (hazard ratio 0.26, 95% CI 0.19-0.35) and 78% (hazard ratio 0.22, 95% CI 0.18-0.27) reduction in risk of testing positive for SARS-CoV-2 for individuals that received the Oxford/AstraZeneca and Pfizer/BioNTech vaccines, respectively, when compared with unvaccinated individuals. A very low proportion of hospital admissions were seen in vaccinated individuals who tested positive for SARS-CoV-2 (288/389,587, 0.07% of all patients vaccinated) providing evidence for vaccination effectiveness after a single dose.

**Conclusions:**

There was no definitive evidence to suggest COVID-19 was transmitted as a result of vaccination hubs during the vaccine administration rollout in North West London, and the risk of contracting COVID-19 or becoming hospitalized after vaccination has been demonstrated to be low in the vaccinated population. This study provides further evidence that a single dose of either the Pfizer/BioNTech vaccine or the Oxford/AstraZeneca vaccine is effective at reducing the risk of testing positive for COVID-19 up to 60 days across all age groups, ethnic groups, and risk categories in an urban UK population.

## Introduction

### Background

On March 11, 2020, the World Health Organization declared the novel coronavirus, SARS-CoV-2 that causes COVID-19, as a pandemic with governments worldwide implementing restrictive measures to slow the spread of the virus and prompting an international effort to develop an effective vaccine [1]. Development of a COVID-19 vaccine by a partnership of BioNTech and Pfizer had commenced on January 10, 2020, following the publication of the SARS-CoV-2 genetic sequencing data, and on December 2, 2020, the United Kingdom became the first country to approve a COVID-19 vaccine after regulators granted emergency use authorization to BNT162b2 mRNA produced by Pfizer and BioNTech following the publication of results of the phase 3 trials [2,3]. The UK mass vaccination program commenced on December 8, 2020 [2]. By December 30, 2020, the ChAdOx1 nCoV-19 adenoviral vaccine, developed by Oxford University/AstraZeneca, was granted regulatory approval by the Medicines and Healthcare Products Regulatory Agency (MHRA), and its use was included in the UK vaccination program [2,4]. The Moderna vaccine was the third COVID-19 vaccine to be approved for use by the MHRA on January 8, 2021, and further vaccines are in development and awaiting approval for use [1]. The Joint Committee on Vaccination and Immunisation established the strategy, on behalf of the Government, for the rapid distribution of a first dose of a vaccine to groups of the population deemed to be most vulnerable to severe COVID-19 infection [5]. By February 26, 2021, 29% of the UK population had received at least one dose of an approved COVID-19 vaccine [6]. The Joint Committee on Vaccination and Immunisation–stated target was to have offered a first vaccine dose to everyone in priority groups one, two, three, and four by February 15, 2021 [7].

Anticipated vaccination coverage of priority groups has been reduced by vaccine hesitancy, which is present in the United Kingdom and Continental European populations alike [8,9]. To ensure the sufficient and rapid uptake of the offered vaccination program, identifying and addressing vaccination hesitancy and resistance (ie, the positions where one is unsure about taking a vaccine or where one is absolutely against taking a vaccine) is essential [10]. The use of vaccination centers has been reported to increase vaccine hesitancy, possibly due to fear of transmission, but is the only feasible way of administering large numbers of vaccinations rapidly given logistical and cold storage constraints [9]. Identifying and understanding COVID-19 vaccine hesitancy within distinct populations may aid future public health messaging.

Real-world data supporting the effectiveness of the vaccination strategy in the UK population is needed to guide health policy. This real-word data-driven evidence study of the UK COVID-19 vaccination program in the North West London (NWL) population used a unique data set established as part of the Gold Command COVID-19 response in NWL [11], which included the pre-established Whole System Integrated Care (WSIC) data collated for the purposes of population health in the sector.

WSIC is an innovative data sharing initiative by the NWL Collaboration of Clinical Commissioning Groups (CCGs) and has been designed to improve data sharing and interoperability [12,13]. WSIC dashboards link provider data from four acute, two mental health, and two community Trusts across eight CCGs; social care data from eight boroughs; and 360 general practitioner (GP) practices to generate an integrated care record for direct patient benefit. The COVID-19 dashboard allows access to data on vaccination and SARS-CoV-2 testing within minutes or hours of the data being recorded in source data systems. The vaccination dashboard uses GP clinical systems (SystemOne, eMIS), pathology laboratories (NWL Pathology and The Doctor’s Laboratory), national COVID-19 test results, and daily COVID-19 situation reports from the Northwest London secondary care organizations.

### Aims and Objectives

The aim of this study is to assess the early vaccine administration coverage and vaccine effectiveness and outcome data across an integrated care system of eight CCGs leveraging a unique population-level care data set.

The study objectives were:

- To describe vaccination coverage across NWL CCGs and identify subgroups according to sociodemographic factors and including where vaccination offer was declined
- To investigate the impact of vaccine administration on possible virus transmission by assessing rates of positive testing after vaccination and to examine the potential importance of continued isolation following the delivery of a single dose of a COVID-19 vaccine
- To assess the early effectiveness of COVID-19 vaccination over a 10-week follow-up period stratified across population subgroups and by vaccine type, and compared with rates of SARS-CoV-2 positive testing rates in the nonvaccinated population

## Methods

### Study Design

The study was a retrospective cohort design. Data were captured to support the NWL response to the COVID-19 pandemic on behalf of NWL Gold Command as part of Whole Systems Integrated Care. Anonymized data covering vaccinated and unvaccinated individuals from NWL were accessed in the iCARE (Imperial Clinical Analytics Research and Evaluation) system [11] for analysis.

### Participants and Setting

All adults older than 16 years, eligible to be offered a COVID-19 vaccine and registered with a GP or with a resident postcode in the NWL catchment area were included in the analysis. The eligible population was considered as a static group over the study period based on data available on February 24, 2021.

Vaccinated individuals were defined as persons receiving a vaccine within the NWL vaccine program time period, considered December 8, 2020, to February 15, 2021, inclusive. Vaccination status was provided either directly via acute hubs or via GP electronic patient record systems via primary care hubs. The unvaccinated group were considered those that had not received a vaccine during the same NWL vaccine program time period.

Individuals were counted as declining a vaccine if they indicated that they did not want a vaccine to their GP and did not then receive a vaccine. Rates of declining vaccination were calculated using the denominator of those who received a vaccine or those that declined a vaccine. Individuals who initially declined vaccination but then were vaccinated after February 15, 2021, and before February 24, 2021, were not included as vaccinated.

Follow-up analysis included data until February 24, 2021 (inclusive), for both groups, allowing over a week of follow up for all individuals.

### Variables

The analysis data set was created through the combination of data from GP primary care systems, including SARS-CoV-2 test results (pillar 2), vaccination status and type, contraindications to COVID-19 vaccination, vaccination decline, age, gender, ethnic group, clinically extremely vulnerable status, and decile of deprivation; social care data sets, including care home and housebound status; pathology laboratory data, including SARS-CoV-2 test results obtained from NWL Pathology, The Doctors Laboratory (pillar 1), and national SARS-CoV-2 test results; and NWL acute Trust patient-level situation reports, including admission and discharge dates.

Risk groups were defined in WSIC (based on the Joint Committee on Vaccination and Immunisation priority cohorts); these were based primarily on individuals in care homes, then those classed as clinically extremely vulnerable, and then on age groups of individuals. Therefore, in the analysis where risk groups were used, it should be assumed that the care home and clinical extremely vulnerable can be of any age. Those in care homes were predominantly, although not exclusively, older individuals. Frontline key worker status could not be identified from the data available and therefore could not be analyzed separately.

Outcomes measured were the date of result for the first positive swab for all individuals (lateral flow test results were excluded), and results included tests from pillar one and two [2]. All nonpositive (negative, inconclusive, and error) were grouped as nonpositive results, with the assumption that all nonnegative tests would be followed with a second test, and these positive results would be included if returned. The denominator for the week-on-week population groups was calculated based on the number of individuals with follow-up data available up to the start of each weekly time period and who had not previously tested positive. Testing rates pre- and postvaccination were examined to identify if changes in individual’s likelihood of being tested could impact changes in levels of positive SARS-CoV-2 testing.

Secondary outcomes of hospitalization due to COVID-19 were measured as vaccinated patients admitted to the hospital who had tested positive for SARS-CoV-2 prior to admission or recorded a positive result in the first 7 days of inpatient stay [14]. All secondary care data were recorded from situation reports data submitted by NWL acute Trusts. This does not include diagnosis data or reason for admission to hospital.

Individuals that received Moderna vaccines (n=3) were excluded from analysis comparing vaccination types due to insufficient numbers. Patients who died (all cause) between December 8, 2020, and February 24, 2021, were excluded from the main analysis and included in a subanalysis, as date of death in the upstream systems is updated variably and therefore likely to be an underestimate.

### Identification of Bias

Variations in prevalence of COVID-19 in the population across the timescale of this longitudinal study may alter the rate of positive testing in both the vaccinated and unvaccinated groups. To address these potential confounding factors, prevalence of positivity in the background population and the rate of vaccination delivery were compared.

Unequal use of vaccine type across risk cohorts could make a direct comparison of vaccine outcome data unreliable. We have stated the delivery rates of vaccination types and adjusted denominators appropriately for return to follow up.

Individuals with COVID-19 that did not test positive (untested or asymptomatic) would be included in the COVID-19 negative population. It was assumed that individuals not testing positive were negative. The data set does not include lateral flow positive tests, which may be more represented in key frontline workers, although frontline workers make up a minority number of the overall NWL population.

The cause of hospital admission of patients was not provided in the NWL acute Trust situation reports and therefore was not available. It was assumed that a COVID-19–related admission would include any patient testing positive in the period prior to an admission or within 7 days of an admission, as per the Public Health England definition [14]. It was not appropriate to compare hospital admissions between vaccinated and unvaccinated groups, as the vaccination program has targeted the most high-risk individuals, with therefore a presumed higher risk of admission, due to comorbidities.

### Statistical Analysis Methods

Known missing data included vaccination type for <1% of vaccinated individuals; these data were included in analysis of overall vaccinations but excluded from vaccination type breakdowns (unless indicated).

Pearson product-moment correlations were used to measure the correlation between individuals declining a vaccination and socioeconomic deprivation status. Index of multiple deprivation (IMD) deciles are the official measure of deprivation in the United Kingdom [15] and are assigned to individuals based on home postcode.

Vaccine effect estimation was calculated using time-to-event analysis. Cumulative SARS-CoV-2 positive results were graphically displayed using Kaplan-Meier curves stratified by vaccination status. Follow-up time commenced on December 8, 2020, which was the start of the vaccination program, for those unvaccinated and commenced on the day of vaccination for those vaccinated. All patients were followed up until a positive SARS-CoV-2 test result or censoring on February 24, 2021. As a positive SARS-CoV-2 test result is a nonfatal event, we used mortality as a competing risk (ie, the individual died before having the outcome event).

Multivariable Cox regression analysis was used to investigate whether vaccination independently predicted having a SARS-CoV-2–positive swab during follow-up compared to unvaccinated individuals, after adjusting for age, gender, ethnicity, IMD, and vaccine manufacturer. We performed a time-dependent Cox regression analysis of vaccination effectiveness on SARS-CoV-2 positivity during follow-up in all individuals up to 28 days post vaccination in the following time intervals: 0-7, 8-14, 15-21, and 22-28 days. Analyses were performed with the use of R software, version 4.0.1 (R Foundation for Statistical Computing).

### Ethics

This study was undertaken within a research database that was given favorable ethics approval by the West Midlands Solihull Research Ethics Committee (reference 18/WM/0323; IRAS project ID 252449). All data used in this paper were fully anonymized before analysis.

## Results

### Vaccination Coverage

In NWL, 2,183,939 individuals were eligible to receive a COVID-19 vaccine. A total of 1,059,280 (48.5%) were female; 930,877 (42.6%) were White; 529,492 (24.2%) were Asian or Asian British; 166,011 (7.6%) were Black or Black British; 60,483 (2.8%) were mixed race; and 189,877 (8.7%) were other ethnic groups. There was no ethnicity recorded for 307,099 (14.1%) individuals.

**Figure 1:**
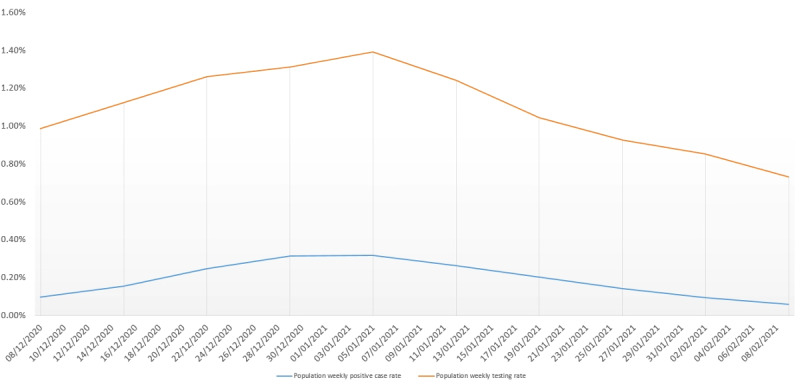
Weekly person SARS-CoV-2 testing rate compared to weekly positive case rate in population eligible for vaccination over duration of study.

The week-on-week testing rate as a proportion of the overall NWL eligible population reached a peak of 1.39% (n=30,396 tested persons) of the population by the week commencing January 5, 2021 (Figure 1). After this, it fell to 0.73% (n=15,946) of the population in the week commencing February 9, 2021. Eligible population prevalence of positive cases in a week peaked in early January at 0.32% (n=6805 cases) and then fell steadily each week to 0.06% (n=1017 cases) of the population in the week commencing February 9, 2021, with the average across all weeks in the study being 0.19%.

**Figure 2:**
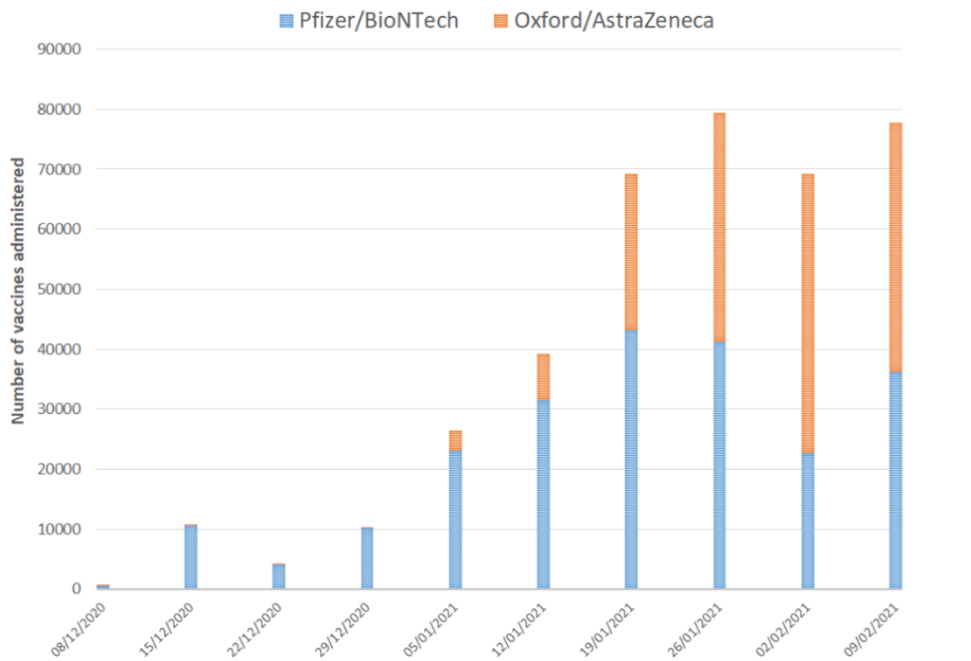
Number of first dose vaccinations given per week in the eligible population from December 8, 2020, during the 10-week study period (numbers of vaccines administered defined as all first dose vaccines delivered within the 7-day period from the weekly start date indicated).

By February 15, 2021, 389,587 (17.84%) individuals had received at least one dose of a COVID-19 vaccine. Vaccination administration notably increased from early January 2021 with the period between January 5 and February 15, 2021, accounting for 363,304 (93.25%) of the total 389,587 vaccines administered (Figure 2). The number of Oxford/AstraZeneca vaccines administered started to reach parity with Pfizer/BioNTech by mid-January. In the NWL vaccination program time period overall, 223,201 (57.29%) Pfizer/BioNTech and 163,452 (41.96%) Oxford/AstraZeneca vaccines were administered. Pfizer was administered to the majority of individuals aged 16-49 years (n=47,817/71,585, 66.80%), 75-79 years (n=25,348/41,057, 61.74%), and 80 years or older (n=42,090/58,116, 72.42%). In those aged 50-74 years, Pfizer and AstraZeneca were administered with similar proportions (Pfizer: n=89,419/174,115, 51.3%); AstraZeneca was administered to the majority of care home residents (n=3822/5186, 73.7%) and in the clinically extremely vulnerable (n=21,014/38,532, 54.5%).

**Figure 3:**
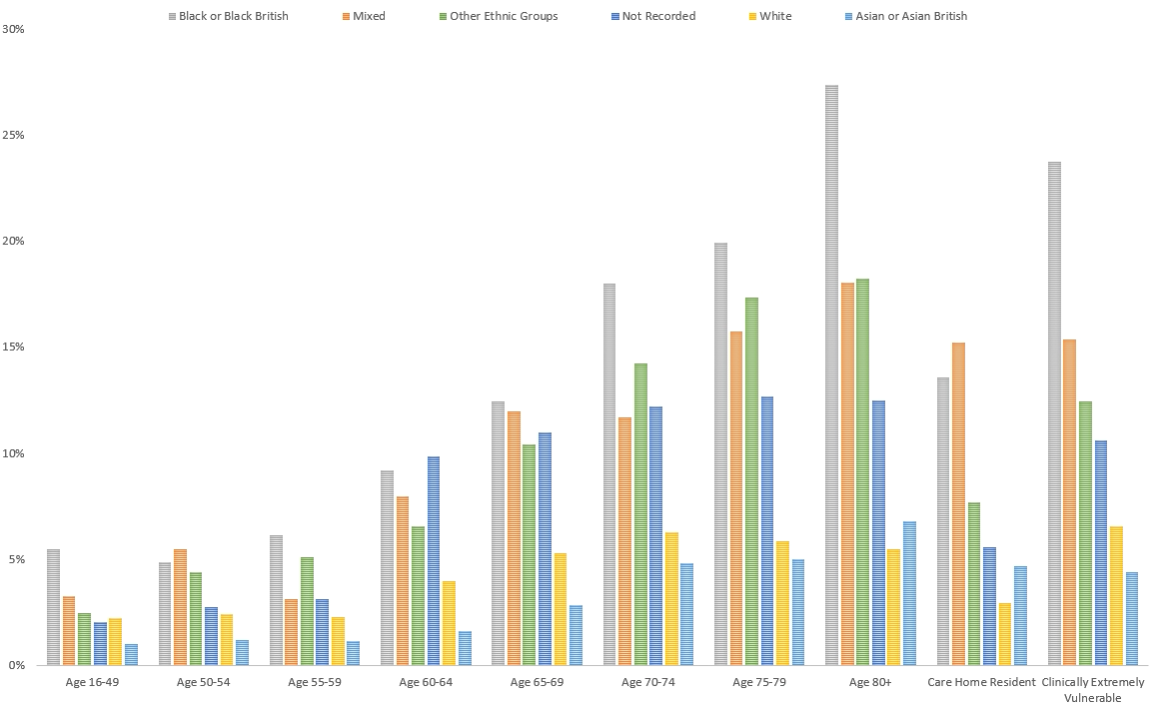
The percentage of population declining vaccination across Whole System Integrated Care risk categories according to ethnicity during the study period.

During the NWL vaccine program time period, 413,919 individuals were offered a vaccine and 24,332 (5.88%) people declined and did not receive a vaccination. In the vaccinated group, 2957 patients had initially declined but subsequently went on to receive a vaccination, indicating a hesitancy rate of 0.71% (where an individual is initially unsure about taking a vaccine) over the study period. Over the study time period, the rate of declining a vaccination across all Black, Asian, and minority ethnic groups was 6.39% (11,528/180,210) compared with the White group at 4.92% (9788/187,090). Black or Black British individuals had the highest rate of declining a vaccine at 16.14% (4337/26,870). Mixed ethnicity groups’ vaccine declining rate was 10.39% (895/8613). In the Asian and Asian British groups, the rate of declining vaccines was the lowest at 3.21% (3867/120,291). Other ethnic groups’ declination rate was 9.95% (2429/24,409), and the ethnicity unrecorded group declination rate was 8.52% (3016/35,419). Within the Black or Black British individuals, the highest rates of declining vaccination during the study period were seen in those 80 years or older or those clinically extremely vulnerable at 27.58% (1384/5018) and 23.97% (940/3911), respectively (Figure 3).

Overall during the study period, there were similar rates of declining vaccination between gender (female: 13,595/229,732, 5.92%; male: 10,736/184,180, 5.83%). Younger males had a higher rate of declining vaccination than younger females (younger than 65 years, female: 1817/83,872, 2.17%; younger than 65 years, male: 1903/60,221, 3.16%). Conversely, older females had a higher rate of declining vaccination than older males (65 years or older, female: 9594/120,8327, 0.94%; 65 years or older, male: 7186/101,438, 7.08%). There was a strong negative association between deprivation and rate of declining vaccination (*r*=–0.94; *P*=.002) with 13.5% (1980/14,571) of individuals declining vaccination in the most deprived postcodes compared to 0.98% (869/9609) in the least deprived postcodes. For individuals living in the most deprived areas (bottom decile), those with the highest rates of vaccine decline were older than 70 years (70-74 years: 344/1963, 17.52%; 75-80 years: 275/1448, 18.99%; 80 years or older: 524/2022, 25.91%), clinically extremely vulnerable (377/1967, 19.17%), and from Black and Black British (337/1967, 25.79%) communities.

### Impact of Vaccine Administration on Possible Virus Transmission

In the first 6 days after vaccination, 344 of 389,587 (0.09%) individuals tested positive for SARS-CoV-2. The rate increased to 0.13% (525/389,243) between days 7 and 13, before then gradually falling week by week (Table 1). By week 7, fewer than 20 persons were testing positive each week (weekly rate≤0.05% week 5 onward). Over the same time period, no appreciable decrease in the amount of testing of the vaccinated population was observed, indicating that this was not an effect linked to a reduction in levels of testing in individuals after vaccination.

Care home residents and housebound individuals had a higher rate of positivity in the second week post vaccination at 0.35% (55/15,742) compared with the non–care home or housebound group at 0.13% (525/389,249; Table 1). After the second week, the rate of positivity decreased, although it took until week 5 to reach less than 0.1%. There was a trend to suggest the rate of positivity decrease week on week was slower when compared with the non–care home and housebound group, but absolute numbers of positive cases in care homes and housebound individuals were very low. Overall, the mean age of care home and housebound residents was 80.6 years.

**Table 1:** Absolute numbers of first positive SARS-CoV-2 tests per week after day of vaccination and weekly rates of testing based on individuals available for follow-up (excluding previously positive cases).^a^

Vaccinations	Days after vaccination										
	<7 (week 1)	7-13 (week 2)	14-20 (week 3)	21-27 (week 4)	28-34 (week 5)	35-41 (week 6)	42-48 (week 7)	49-55 (week 8)	56-62 (week 9)	63-69 (week 10)	≥70 (≥week 11)
Vaccinated individuals time to first positive test after vaccination, n	344	525	332	147	87	48	16	13	11	<5^b^	0
Total vaccinated population completed to period of follow-up (excluding previously positive patients), n	389,587	389,243	330,523	261,447	184,847	111,555	62,283	31,757	20,097	14,200	2519
First positive individuals by population completed to follow-up time to first positive (not previously positive), %	0.09	0.13	0.10	0.06	0.05	0.04	0.03	0.04	0.05	0.01	0.00
Vaccinated individuals (excluding care home or housebound residents) time to first positive test after vaccination, n	319	470	284	129	71	46	13	10	9	<5	0
Total vaccinated population (excluding care home and housebound residents) completed to period of follow-up (excluding previously positive patients), n	373,820	373,501	315,666	248,136	173,336	105,834	59,574	30,674	19,338	13,763	2431
First positive individuals by population completed to follow-up time to first positive (not previously positive; excluding care home and housebound), %	0.09	0.13	0.09	0.05	0.04	0.04	0.02	0.03	0.05	0.01	0.00
Vaccinated care home or housebound individuals time to first positive test after vaccination, n	25	55	48	18	16	<5	<5	<5	<5	0	0
Total vaccinated care home or housebound population completed to period of follow-up (excluding previously positive patients), n	15,767	15,742	14,860	13,317	11,556	5770	2749	1090	771	443	92
First positive care home or housebound individuals by population completed to follow-up time to first positive (not previously positive), %	0.16	0.35	0.32	0.14	0.14	0.03	0.11	0.28	0.26	0.00	0.00

^a^Rates are stratified by individuals in care homes or housebound and those in the rest of the vaccinated population.

^b^Low numbers (1-4) have been replaced with <5.

**Figure 4:**
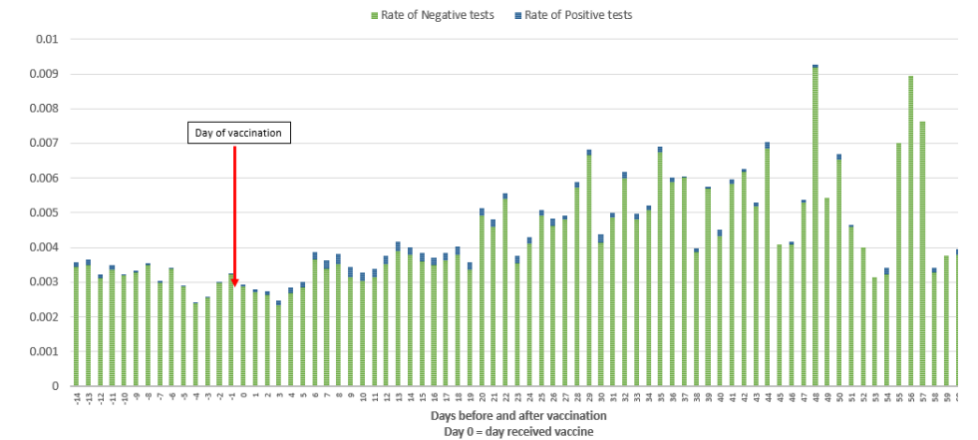
The proportion of all SARS-CoV-2 tests in the vaccinated population (not limited to the first positive) each day following administration of their first vaccine dose, based on the number of individuals available for follow-up to the end of the study period, split by positive and nonpositive results.

The testing rate was lowest in the 3- to 4-day period either side of the day of vaccination (Figure 4). After vaccination, the testing rate increased and remained, on average, higher until day 60. Data after day 60 was not included at the daily level due to low numbers. The reduction in positive test results after vaccination could not be attributed to overall reduction in testing over time.

**Figure 5:**
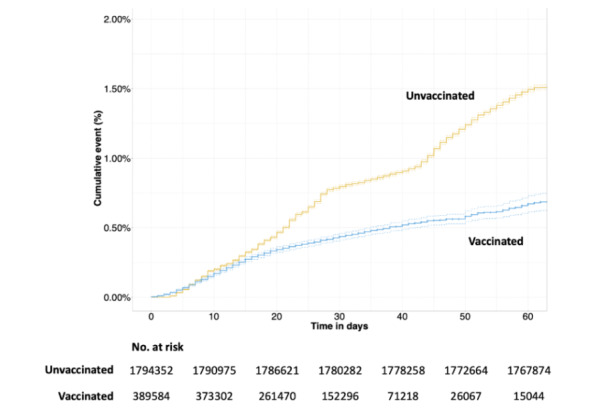
Cumulative event rate with a positive SARS-CoV-2 test result in the vaccinated and unvaccinated groups available for follow-up. Numbers at risk are calculated at 10-day intervals. Dotted lines depict 95% CIs.

In summary, Table 1 shows that infections decrease from day 14 post vaccination to rates that are lower than, or equivalent to, the population weekly levels (Figure 1), and these decreases are not a result of a reduction in testing post vaccination (Figure 4). The risk of COVID-19 infection rate was lower in the vaccinated population than the unvaccinated population (Figure 5). The time to testing positive in the vaccinated group compared with the unvaccinated group was similar until day 15 post vaccination when the groups appear to diverge (Figure 5).

### COVID-19 Vaccination Effectiveness

Vaccination effectiveness was measured according to the rates and hazard ratios (HRs) of testing positive post vaccination compared to the unvaccinated population. In individuals that tested positive post vaccination, levels of hospital admissions due to COVID-19 were measured. Of the eligible vaccination cohort, the average length of follow-up post vaccination was 29 days, with a range of follow-up being 10 to 79 days. The time to testing positive in the vaccinated group compared with the unvaccinated groups was similar until day 15 post vaccination when the groups appear to diverge, with a smaller cumulative risk in the vaccinated population of testing positive over time (Figure 5).

**Figure 6:**
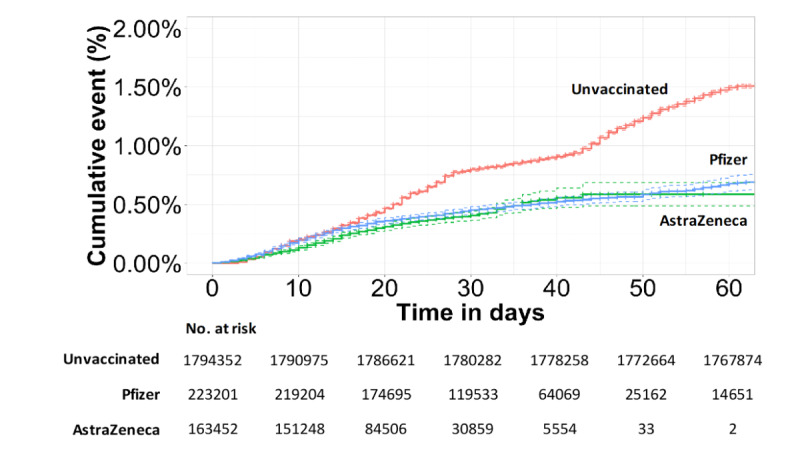
Cumulative event rate of testing positive comparing Pfizer and AstraZeneca vaccination groups to the unvaccinated group available for follow-up. Numbers at risk are calculated at 10-day intervals. Vaccination type was not available for 2934 patients. Dotted lines depict 95% CIs.

At 28 days post vaccination, there was a 74% (HR 0.26, 95% CI 0.19-0.35) and 78% (HR 0.22, 95% CI 0.18-0.27) reduction in risk of testing positive for COVID-19 for individuals that received the Oxford/ AstraZeneca and Pfizer/BioNTech vaccines, respectively, when compared with unvaccinated individuals (Table 2). There was a lack of significant follow-up data in the Oxford/AstraZeneca group to make a meaningful comparison past 28 days; therefore, these results are not displayed with HRs in Table 2. As a reflection of differences in availability of each of the vaccines, patients who were administered the Pfizer vaccination had longer follow-up to those who were administered the AstraZeneca vaccine (Figure 6). There were no differences in SARS-CoV-2–positive event rates comparing people who had the Pfizer and Oxford/AstraZeneca vaccinations (Figure 6).

**Table 2:** Time-dependent Cox regression analysis of vaccination effect each week following delivery on SARS-CoV-2 positivity during follow-up in all individuals up to 28 days post vaccination.

Week period (days)	No vaccination	Oxford/AstraZeneca			Pfizer/BioNTech	
		Hazard ratio (95% CI)	*P* value	Hazard ratio (95% CI)		*P* value
0-7	1.0 (Reference)	0.71 (0.60-0.84)	<.001	1.03 (0.91-1.17)		.65
8-14	1.0 (Reference)	0.68 (0.59-0.80)	<.001	0.90 (0.80-1.00)		.06
15-21	1.0 (Reference)	0.59 (0.49-0.71)	<.001	0.42 (0.36-0.50)		<.001
22-28	1.0 (Reference)	0.26 (0.19-0.35)	<.001	0.22 (0.18-0.27)		<.001

**Figure 7:**
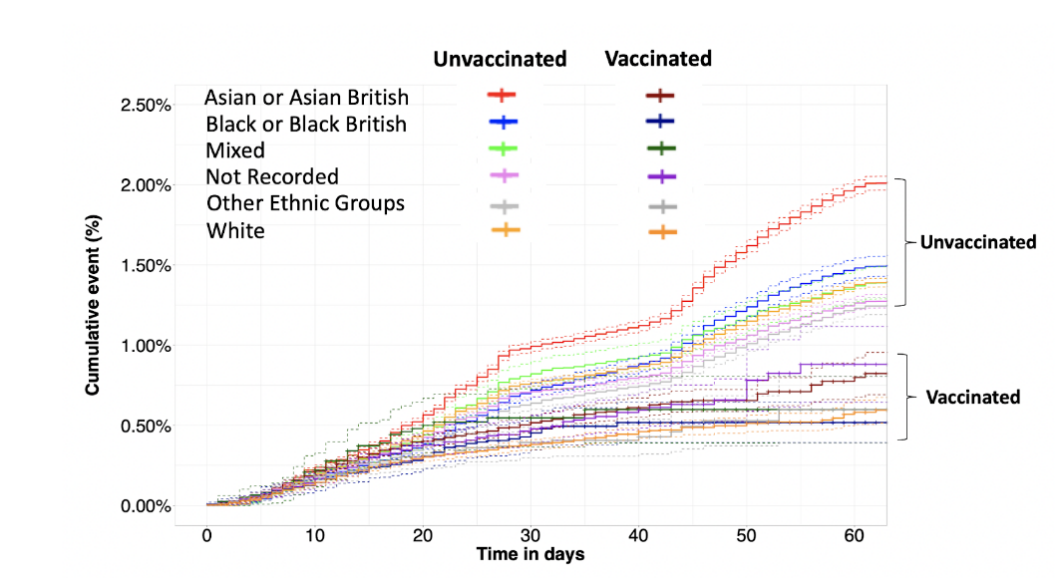
Cumulative event rate of testing for SARS-CoV-2 positive in the vaccinated and unvaccinated groups available for follow-up, stratified by ethnicity. Dotted lines depict 95% CIs.

Unvaccinated care home residents were four times as likely compared with individuals aged 16-49 to test positive (HR 4.05, 95% CI 3.48-4.71). Unvaccinated Asian or British Asian individuals had a multivariable adjusted HR of 1.45 (95% CI 1.41-1.49) of testing positive by 60 days compared to the White group (Multimedia Appendix 1). All ethnic groups benefited from vaccination, with the greatest reduction in risk due to vaccination seen in Asian or Asian British individuals (Figure 7).

Unvaccinated men were less likely to test positive within 60 days than women (HR 0.89, 95% CI 0.86-0.91; Multimedia Appendix 1); however, there was no significant difference between the genders in the vaccinated population (Table 3). There were no significant differences in HRs associated with a positive result with vaccination across ethnicities, IMD decile groups, or gender. Significant differences in HRs show that infections in older age groups (65-69 years, 70-74 years, 75-80 years, and 80 years or older) and in clinically extremely vulnerable were present, showing these groups are significantly less likely to be infected post vaccination, indicating vaccine effectiveness in the oldest population groups (Table 3).

In total, 288 vaccinated individuals were admitted to hospital post vaccination who tested positive for SARS-CoV-2 after vaccination and before (or up to 7 days into) their inpatient stay; this accounted for only 0.07% (288/389,587) of vaccinated individuals. Of these patients, 54% (n=155) were admitted before day 14 after vaccination. Admission rates of vaccinated individuals available to follow up peaked at 0.03% (n=102) in days 7 to 13 after vaccination and reduced to 0.01% (n≤5) or lower from days 28 to 34 after vaccination.

Between December 8, 2020, and February 24, 2021, there were a total of 441 all-cause deaths, which comprised 161 (36.5%) and 280 (64.5%) in the vaccinated and unvaccinated groups, respectively. Of the 161 deaths in the vaccinated group, 18 (11.2%) had a positive SARS-CoV-2 test in the 28 days preceding death (1 in 21,739 of all vaccinated patients). Of the 280 deaths in the unvaccinated group, 68 (24.3%) had a positive SARS-CoV-2 test in the 28 days preceding death (1 in 556 of all unvaccinated patients).

**Table 3:** Multivariable Cox regression analysis showing hazard ratio of a positive SARS-CoV-2 result during follow-up with vaccination in all patients and across different age, ethnic, gender, and IMD decile groups up to day 60 post vaccination.^a^

Variables		Hazard ratio (95% CI)		*P* value
**Vaccination**				
	No vaccination (reference)	1	N/A^b^	
	Vaccination	0.64 (0.43-0.95)	.03	
**Age (years)**				
	16-49 (reference)	1	N/A	
	50-54	0.75 (0.58-0.98)	.03	
	55-59	0.82 (0.64-1.06)	.13	
	60-64	0.79 (0.61-1.02)	.07	
	65-69	0.41 (0.32-0.54)	<.001	
	70-74	0.26 (0.20-0.33)	<.001	
	75-79	0.29 (0.23-0.38)	<.001	
	≥80	0.29 (0.24-0.36)	<.001	
Care home resident		0.76 (0.56-1.05)		.13
Clinically extremely vulnerable		0.30 (0.24-0.38)		<.001
**Ethnicity**				
	White (reference)	1	N/A	
	Asian or British Asian	0.91 (0.80-1.02)	.11	
	Black or Black British	0.98 (0.77-1.25)	.89	
	Mixed	1.29 (0.91-1.82)	.15	
	Other ethnic groups	1.06 (0.83-1.35)	.65	
**Gender**				
	Female (reference)	1	N/A	
	Male	1.02 (0.91-1.15)	.69	
**IMD^c^ decile**				
	1 (reference)	1	N/A	
	2	1.03 (0.74-1.43)	.87	
	3	1.11 (0.82-1.51)	.49	
	4	0.97 (0.71-1.32)	.83	
	5	1.08 (0.79-1.48)	.62	
	6	1.04 (0.76-1.42)	.79	
	7	0.80 (0.58-1.12)	.24	
	8	0.84 (0.59-1.20)	.33	
	9	0.93 (0.65-1.34)	.72	
	10	0.83 (0.56-1.21)	.33	

^a^Cox regression model included an interaction term between having the vaccination and individual patient groups (age, ethnicity, gender, IMD decile).

^b^N/A: not applicable.

^c^IMD: index of multiple deprivation.

## Discussion

### Principal Results

By February 15, 2021, the NWL vaccination program had vaccinated 17.84% (389,587/2,183,939) of the eligible population, according to priority, with at least one dose of a COVID-19 vaccine over a 10-week period, commencing December 8, 2020. Understanding and addressing vaccine hesitancy, across the population offered a vaccine, represents an important improvement opportunity to maximize widespread population vaccination coverage; in this study, 5.88% (24,332/413919) of the NWL eligible population declined a vaccine. Rates of vaccine decline within Black and Black British groups were three times greater (16.14%, 4337/26,870) than the White population. A quarter of Black and Black British individuals who were 80 years or older, or were clinically extremely vulnerable (27.58% and 23.97%, respectively) declined the vaccine. This finding is supported by similar reports examining vaccine hesitancy [16]. There was a strong negative correlation between deprivation score and vaccine hesitancy; individuals in the most deprived areas declined vaccinations at a rate 13 times higher than those in the most affluent areas. Overall across NWL, the highest rates of vaccine decline were seen in older adults and Black British people living in the most deprived areas. The causes for this were not assessed by this study but highlights an important area of focus for quality improvement, public and societal engagement, and outreach initiatives to improve vaccination coverage across all population groups, especially in relation to findings that indicate vaccine effectiveness.

As previous studies have shown, this data supports the strategy of prioritizing the older adult and care home residents, as unvaccinated care home residents were four times as likely to test positive (HR 4.05, 95% CI 3.48-4.71) compared with individuals aged 16-49 years. There is further evidence of differing susceptibility to COVID-19 across sociodemographic groups, which could support further vaccine prioritization to those who would benefit most; unvaccinated Asian and Asian British individuals were at increased risk of testing positive for SARS-CoV-2 compared to the White population (HR 1.45, 95% CI 1.41-1.49), and unvaccinated women more likely to test positive in 60 days than men (male HR 0.89, 95% CI 0.86-0.91).

The incubation period to develop symptoms indicative of COVID-19 is on average 5 to 6 days but can be as long as 14 days [5,7]. This means that the majority of transmission at the point of vaccination should be detected and confirmed by positive test results within 14 days of vaccination. The rate of positive COVID-19 cases in the second week (days 7-13) after receiving a vaccine at a vaccination hub or via a roving team for care home and housebound individuals, peaked at 0.13% (525/389,243). Although this was higher than 0.09% (344/389,587) recorded in days 1 to 6, it was lower than the average weekly person testing positive rate recorded in the total population at 0.19% (average weekly 4112/2,183,503) This supports the conclusion that the act of vaccine delivery in NWL did not increase SARS-CoV-2 transmission above that already seen in the background population. Despite overall low levels of positive testing in the vaccinated group, however, the increase in positive tests recorded in days 7 to 13 after vaccination do suggest some potential for increased SARS-CoV-2 transmission at or after the time of vaccination. It is impossible to identify and separate out several possible contributors to this, including in the days postvaccination individuals were more liberal with isolation and social distancing measures before immunity resulting from vaccination had become effective, some transmission of SARS-CoV-2 occurring at time of vaccine administration, or individuals were asymptomatic but infected when attending for vaccination. Certainly, regarding the latter, there is some evidence to support this, as a number of individuals tested positive within 5 days of attending for vaccination (Figure 4).

In the care home residents or housebound individuals, the rise in positive case rate in the second week post vaccination was greater than that of the rest of the vaccinated population (55/15,742, 0.35% compared to 525/389,243, 0.13%) in non–care home and housebound individuals. This higher rate needs to be interpreted within the context of physically frail groups having innate vulnerability to SARS-CoV-2 transmission [17]. Equally, it is not possible to determine the contribution of postvaccination easing of social distancing and isolation measures prior to the vaccination generating an immune response that provides effective protection. There is also some evidence that the time for older adults to develop effective immunity takes longer than the younger population [18]. This is supported by a trend suggesting the rate of positivity decreases week on week more slowly when compared with the non–care home and housebound group, but absolute numbers of positive cases in care homes and housebound individuals were very low. These results highlight the importance of maintaining physical COVID-19 restriction procedures post vaccination, particularly in the first fortnight. Care home residents and housebound individuals may be particularly vulnerable in the immediate period post vaccination, thus, emphasizing the need to maintain social distancing and restricting visitors to care homes to prevent exposure until population prevalence of COVID-19 has fallen to sufficient levels to make transmission unlikely and time has elapsed to allow postvaccination immunity to develop in this higher risk population. The rise in positive case rates seen in the care home population after the seventh week post vaccination (n≤5 of 1090, 0.28%) raises concerns that the immunological effects of the single vaccine dose may be waning in the frail older adult population over time, which could be due to immunosenescence. The significance of this, however, needs to be interpreted within the small numbers completing follow-up in this group (n=1090). Further studies to examine this are required, as it will have implications for timing of second vaccine administration, which may well vary across priority groups.

Overall, in the NWL population, the rate of positive testing in the vaccinated group compared with the unvaccinated group was similar until day 15, whereafter vaccination reduced an individual’s chance of testing positive for COVID-19 beyond 10 weeks of follow-up. The cumulative risk reduction of testing positive for SARS-CoV-2 at 60 days was 36% (HR 0.64, 95% CI 0.43-0.95; *P*=.03) when receiving a single dose of any vaccine. By the fourth week of follow up (days 22-28), there was similar efficacy for vaccination, with a 74% (HR 0.26, 95% CI 0.19-0.35) and 78% (HR 0.22, 95% CI 0.18-0.27) reduction in risk of testing positive for SARS-CoV-2 in the Oxford/AstraZeneca group and Pfizer/BioNTech group, respectively, compared with the unvaccinated population. There were insufficient numbers of individuals with enough follow-up data in the Oxford/AstraZeneca group to power a statistical comparison between vaccine types beyond 28 days.

The reduction in severity of cases is also evident as demonstrated by the low numbers of admissions to hospitals for vaccinated individuals, with admission rates dropping 14 days post vaccination. Further work is required to compare admissions in the vaccinated population and comparable control populations, including for non–COVID-19 reasons. The vaccinated and unvaccinated populations are inherently different, as vaccination was rolled out according to the priority groups first.

### Limitations

This study uses a unique linked data set that provides real-time data for clinical and operational care delivery, especially relevant during the COVID-19 pandemic. This study highlights the use of these data for generating real-world evidence in accordance with translational data analytics, in addition to data collected through prospective clinical trials. The large sample size of over 2 million people receiving 389,587 doses of a vaccine is a strength of the study with a comparatively long follow-up time compared to other studies that have been reported to date. The cost of running an randomized controlled trial of this size would be significant, but equally, outcome measurements from real-world evidence are less robust, and the results must be interpreted accordingly. The lack of robust control groups to compare with the vaccinated population is problematic, but further analysis similar to methods used by Kaura et al [19] on emulating clinical trials using observational data may be able to address these issues. Follow-up time commenced on December 8, 2020, which was the start of the vaccination program, for those unvaccinated and commenced on the day of vaccination for those vaccinated. Further studies are required that match individuals in the vaccinated and unvaccinated groups on a daily or weekly basis to avoid bias due to differential follow-up start times between the vaccinated and unvaccinated groups, with the potential for exposure to different SARS-CoV-2 strains during follow-up. Soon after the vaccination program started, the national decision was made to schedule the administration of the second vaccination doses, for both Oxford/AstraZeneca and Pfizer/BioNTech, for 10 to 12 weeks after the first dose. As the majority of the first dose vaccinations in NWL were completed in the last 10 weeks of the study period, too small a number of the population had received a second dose at time of data extraction, such that no meaningful analysis could be done addressing completion of the two dose vaccine schedule. Hospitalization due to COVID-19 in the vaccinated population was examined but not compared to the unvaccinated population. This was due to the inherent differences in the groups based on the rollout of vaccinations to those at the highest risk first, meaning unvaccinated individuals would not serve as a suitable control.

The low specificity and sensitivity of some testing mechanisms may provide a degree of error, as rates of positive SARS-CoV-2 tests are used to estimate COVID-19 prevalence in the population. Test results available included pillar one and two but not lateral flow test results. No data were collected on COVID-19 symptoms, and so no assessment on the effects of vaccination on COVID-19 symptoms could be made. By capturing only pillars one and two testing data, this study likely misses asymptomatic cases of COVID-19 in the population, underestimating its true rate. Variation in the prevalence of COVID-19 in the population during the study period could impact the results of the study. Declining rates of COVID-19 in the population during the time of maximal vaccine delivery could have amplified the observed effects of the vaccine.

Only SARS-CoV-2–positive results in the vaccinated group were included in this analysis; therefore, we were not able to assess the impact of antibodies developed from previous COVID-19 infection compared with antibodies developed because of vaccination. However, there remain multiple confounders that cannot be determined from the data, namely, unconfirmed infections, asymptomatic positive individuals, and the uncertain length of time that postvaccination immunity persists. The likely dominant SARS-CoV-2 variant in the examined population at time of study was B1.1.7 [20]. Data on SARS-CoV-2 variants were not collected during the study. The study findings therefore may not be comparable in populations with differing dominant SARS-CoV-2 strains.

### Comparison With Prior Work

A reduction in the risk of testing positive became apparent from day 15 after the administration of a single dose of vaccine in our study. This finding is similar to phase three trial [3] data showing a benefit from day 10 to 13 after a first dose in the Pfizer vaccine and from day 18 in a real-world data study [21]. Interim analysis of four randomized controlled trials in Brazil, South Africa, and the United Kingdom examining the safety and the efficacy of the Oxford/AstraZeneca vaccine did not report efficacy data of a first dose before day 21 post vaccination, showing an efficacy of 64.1% (95% CI 50.5-73.9) after 21 days [4]. Our study demonstrated an observable reduction in risk of testing positive before 21 days, with a 29% (95% CI 16%-40%; *P*<.001) and 32% (95% CI 20%-41%; *P*<.001) reduction in the first (days 0-6) and second (days 7-13) week, respectively, after receiving a first dose of Oxford/AstraZeneca (Multimedia Appendix 2).

Our findings show at 22 to 28 days post vaccination there is a 78% (HR 0.22, 95% CI 0.18-0.27) reduction in risk of testing positive for SARS-CoV-2 after a single dose of the Pfizer/BioNTech vaccine in a cohort representative of a UK urban population. This is comparable to real-world evidence in an Israeli population administered the Pfizer/BioNTech vaccine, showing the early effectiveness of a single dose was estimated to be 52% during the first 24 days after vaccination [21], although a reanalysis of the same data by Hunter and Brainard [22] estimated that, by day 24, vaccine effectiveness had reached 90%. The variation in study design may explain differences seen in efficacy, as the Israeli study used the vaccinated population in days 1 to 12 of vaccination as the control group compared to an unvaccinated control group in our study. Hall et al [23] studied the outcomes of the Pfizer/BioNTech vaccine on a cohort of National Health Service (NHS) health care workers as part of the SIREN study with a similar length of follow-up. This prospective cohort study found reduced levels of vaccine coverage in minority groups, especially Black or Black British groups, similar to our findings, even within a health care worker population. A single dose of Pfizer/BioNTech vaccine demonstrated vaccine effectiveness of 72% (95% CI 58-86) 21 days after the first dose, comparable to our findings, although in a purely working age population [23].

Bernal et al [24] are awaiting peer review of their test negative case-control study estimating the effect of vaccination with the BNT162b2 and ChAdOx1 COVID-19 vaccines in an older population 70 years or older in England. Individuals 80 years or older immunized with BNT162b2 vaccine demonstrated an effectiveness of 70% (95% CI 59%-78%) from 28 to 34 days. ChAdOx1 vaccine effects were seen from 14 to 20 days after vaccination, reaching an effectiveness of 60% (95% CI 41%-73%) from 28 to 34 days and further increasing to 73% (95% CI 27%-90%) from day 35 onward. Similar to our findings Bernal et al [24] demonstrated an increased risk of testing positive in the first 14 days after receiving a vaccine, and those 80 years or older were at particular risk in the first 9 days after vaccination (odds ratio up to 1.48, 95% CI 1.23-1.77).

The causes of vaccine decline were not assessed in this study, but predictors of negative attitudes to vaccines both before and during the COVID-19 pandemic have been described previously in the literature, with most common reasons for hesitancy reported as fear of side effects and long-term health effects and lack of trust in vaccines, particularly among Black respondents [25,26]. Groups with higher rates of vaccine decline are also the same groups seen to be at an increased risk of serious complications from COVID-19, highlighting an important area of focus for outreach initiatives [27].

### Conclusions

This study provides further evidence that a single dose of either the Pfizer/BioNTech vaccine or the Oxford/AstraZeneca vaccine is effective at reducing the risk of testing positive for SARS-CoV-2 up to 60 days across all adult age groups, ethnic groups, and risk categories in an urban UK population. There was no difference in effectiveness up to 28 days between the Oxford/AstraZeneca and Pfizer/BioNTech vaccines. In those declining vaccination, higher rates were seen in those living in the most deprived areas and in Black and Black British groups.

There was no definitive evidence to suggest COVID-19 was transmitted as a result of vaccination hubs during the vaccine administration rollout in NWL, and the risk of contracting COVID-19 or becoming hospitalized after vaccination has been demonstrated to be very low in the vaccinated population. Individuals appear to be less susceptible to COVID-19 transmission in the first weeks after receiving a vaccine as compared with the unvaccinated population; however, a clear message reinforcing the need to continue social distancing restrictions post vaccination should be delivered at the time of vaccination and potentially for up to 21 days. There is also evidence to suggest that in the care home and housebound population, the period of social distancing measures should be more carefully adhered to post vaccination, as initial evidence suggests the time to potentially acquire immunity in this group could take longer than in the general population.

## Appendix

Multimedia Appendix 1 Supplementary Table. Multivariable Cox regression analysis showing hazard ratio of a positive SARS-CoV-2 result in the unvaccinated group across different age, ethnic, gender, and index of multiple deprivation decile groups to day 60 post vaccination.

Multimedia Appendix 2 Supplementary Figure. Cumulative event rate of testing SARS-CoV-2–positive over the first 2 weeks from day of vaccination comparing Pfizer and AstraZeneca vaccination groups to the unvaccinated group. Dotted lines depict 95% CIs.

## Abbreviations

CCG: Collaboration of Clinical Commissioning Group
GP: general practitioner
HR: hazard ratio
iCARE: Imperial Clinical Analytics Research and Evaluation
IMD: index of multiple deprivation
MHRA: Medicines and Healthcare Products Regulatory Agency
NHS: National Health Service
NIHR: National Institute for Health Research
NWL: North West London
WSIC: Whole System Integrated Care
