# Supplementary material for: Assessing COVID-19 Vaccine Uptake and Effectiveness Through the North West London Vaccination Program: Retrospective Cohort Study
Source: JMIR Public Health Surveill. 2021 Sep 17;7(9):e30010. doi: 10.2196/30010 (PMC8451961; doi:10.2196/30010)
Supplement: Multimedia Appendix 1 [file publichealth_v7i9e30010_app1.docx]

|  | **Univariable** | | **Multivariable** | | |
| --- | --- | --- | --- | --- | --- |
|  | **HR (95% CI)** | ***P*** | **HR (95% CI)** | ***P*** | |
| Age | | | | |  |
| 16-49 years (reference) | 1 | - | 1 | - | |
| 50-54 years | 1.07 (1.02 - 1.11) | <.001 | 1.12 (1.07 - 1.17) | <.001 | |
| 55-59 years | 1.00 (0.95 - 1.05) | .97 | 1.06 (1.00 - 1.11) | 0.03 | |
| 60-64 years | 0.93 (0.88 - 0.99) | .01 | 1.01 (0.95 - 1.07) | 0.78 | |
| 65-69 years | 0.85 (0.78 - 0.93) | <.001 | 1.06 (0.97 - 1.15) | 0.22 | |
| 70-74 years | 0.73 (0.66 - 0.80) | <.001 | 1.11 (1.01 - 1.23) | 0.04 | |
| 75-79 years | 0.71 (0.63 - 0.79) | <.001 | 1.16 (1.03 - 1.30) | 0.01 | |
| 80+ years | 0.87 (0.80 - 0.94) | <.001 | 1.49 (1.36 - 1.63) | <.001 | |
| Care Home Resident | 2.75 (2.39 - 3.16) | <.001 | 4.05 (3.48 - 4.71) | <.001 | |
| Clinically extremely vulnerable | 1.52 (1.41 - 1.64) | <.001 | 1.83 (1.69 - 1.98) | <.001 | |
| Ethnicity | | | | |  |
| White (reference) | 1 | - | 1 | - | |
| Asian or asian british | 1.44 (1.41 - 1.48) | <.001 | 1.45 (1.41 - 1.49) | <.001 | |
| Black or black british | 1.09 (1.04 - 1.14) | <.001 | 1.01 (0.97 - 1.06) | .64 | |
| Mixed | 1.05 (0.98 - 1.12) | .21 | 1.01 (0.97 - 1.06) | .87 | |
| Other ethnic groups | 0.92 (0.88 - 0.96) | <.001 | 0.89 (0.86 - 0.94) | <.001 | |
| Gender | | | | |  |
| Female (reference) | 1 | - | 1 | - | |
| Male | 0.89 (0.87 - 0.91) | <.001 | 0.89 (0.86 - 0.91) | <.001 | |
| IMD decile | | | | |  |
| 1 (reference) | 1 | - | 1 | - | |
| 2 | 1.05 (0.99 - 1.11) | .10 | 1.02 (0.96 - 1.09) | .51 | |
| 3 | 1.07 (1.01 - 1.13) | .02 | 1.01 (0.95 - 1.07) | .82 | |
| 4 | 1.02 (0.96 - 1.07) | .58 | 0.97 (0.91 - 1.03) | .26 | |
| 5 | 1.04 (0.98 - 1.10) | .22 | 0.97 (0.92 - 1.04) | .40 | |
| 6 | 0.99 (0.94 - 1.05) | .76 | 0.97 (0.91 - 1.03) | .35 | |
| 7 | 0.86 (0.81 - 0.92) | <.001 | 0.86 (0.80 - 0.92) | <.001 | |
| 8 | 0.86 (0.80  - 0.92) | <.001 | 0.84 (0.78 - 0.90) | <.001 | |
| 9 | 0.92 (0.85 - 0.99) | .03 | 0.92 (0.85 - 1.00) | .05 | |
| 10 | 1.05 (0.96 - 1.15) | .27 | 1.03 (0.94 - 1.13) | .54 | |
