# Supplementary figures and images for: Assessing COVID-19 Vaccine Uptake and Effectiveness Through the North West London Vaccination Program: Retrospective Cohort Study
Source: JMIR Public Health Surveill. 2021 Sep 17;7(9):e30010. doi: 10.2196/30010 (PMC8451961; doi:10.2196/30010)

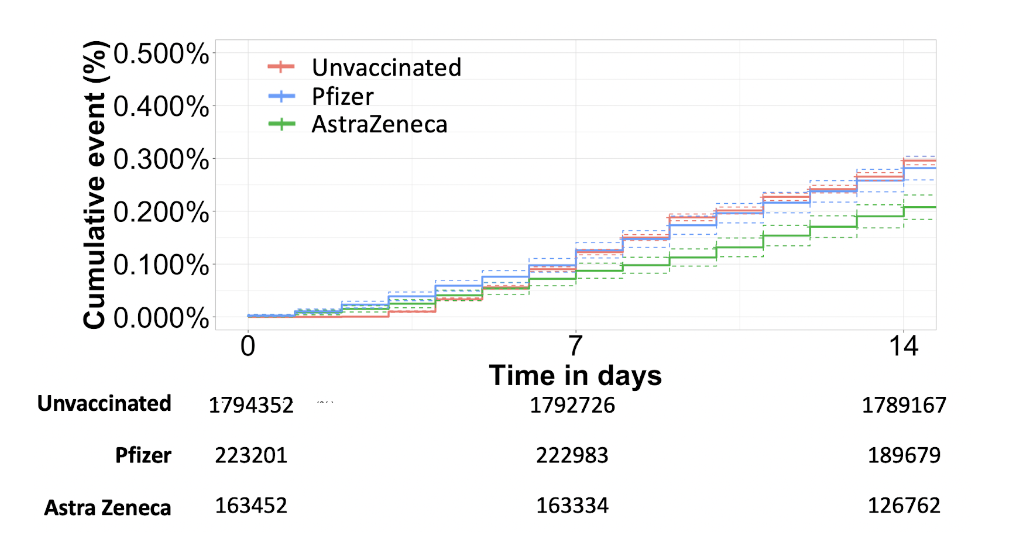

Supplement: Multimedia Appendix 2 [file publichealth_v7i9e30010_app2.png]
